# Effects of transforming growth factor beta-activated kinase 1 (TAK1) on apoptosis of HK-2 cells in the high glucose environment

**DOI:** 10.1080/21655979.2022.2040875

**Published:** 2022-02-21

**Authors:** Yuhan Wang, Li Zhao, DuoJun Qiu, Yonghong Shi, Huijun Duan

**Affiliations:** a Department of Pathology, Hebei Medical University, Shijiazhuang, Hebei, China; b Department of Gastroenterology and Hepatology, Tangshan Gongren Hospital, Tangshan, Hebei, China

**Keywords:** Diabetic nephropathy, TAK1/p38 MAPK/TGF-β1 signaling pathway, reactive oxygen species, apoptosis

## Abstract

To observe the role of transforming growth factor beta-activated kinase 1 (TAK1)/p38 MAPK/TGF-β1 signal pathway plays in oxidative stress and apoptosis in human renal tubular epithelial cells (HK-2) under high glucose induction. HK-2 cells were cultured in high glucose medium with and without TAK1 inhibitor 5Z-7-oxozeaenol. TUNEL and flow cytometry were used to detect cell apoptosis. The protein expression of TAK1, TGF-β1, Bax and Bcl-2 was detected by immunofluorescence. Meanwhile, flow cytometry was used to detect the production of reactive oxygen species (ROS), and MitoSOX staining was performed to detect the production of mitochondrial ROS. Moreover, real-time quantitative PCR and Western blotting was used to measure the expression of TAK1, TGF-β1, NOX1, NOX4 and HO-1, Bax, Bcl-2, p38MAPK, p-p38MAPK and TGF-β1. Results showed that high glucose up-regulated the protein expression of p-TAK1, p-p38 MAPK and TGF-β1, which induced the aggravation of oxidative stress by promoting the production of ROS, thus promote the apoptosis in HK-2 cells. However, addition of 5z −7-oxozeaenol in HK-2 cells reversed all the above functions induced by high glucose. Another experimental result also showed that SB203580, a p38MAPK inhibitor can down-regulated TGF-β1 expression and reduce ROS production, thus alleviate cell apoptosis in TAK1 overexpression group. In summary, high glucose intervention could activate TAK1 and promote apoptosis in HK-2 cells. Inhibition of TAK1 expression could block p38 MAPK/TGF-β1 signaling pathway and reduce ROS production and oxidative stress, which may be one of the signal pathways of TAK1 to reduce apoptosis of HK-2 cells induced by high glucose.

**Abbreviations**: DN, Diabetic nephropathy; TAK1, transforming growth factor β-activated kinase-1; TGF-β, transforming growth factor-β; NG, normal glucose; HG, high glucose; p38 MAPK, p38 mitogen-activated protein kinase; ROS, reactive oxygen species.

## Graphical Abstract

**Figure:**
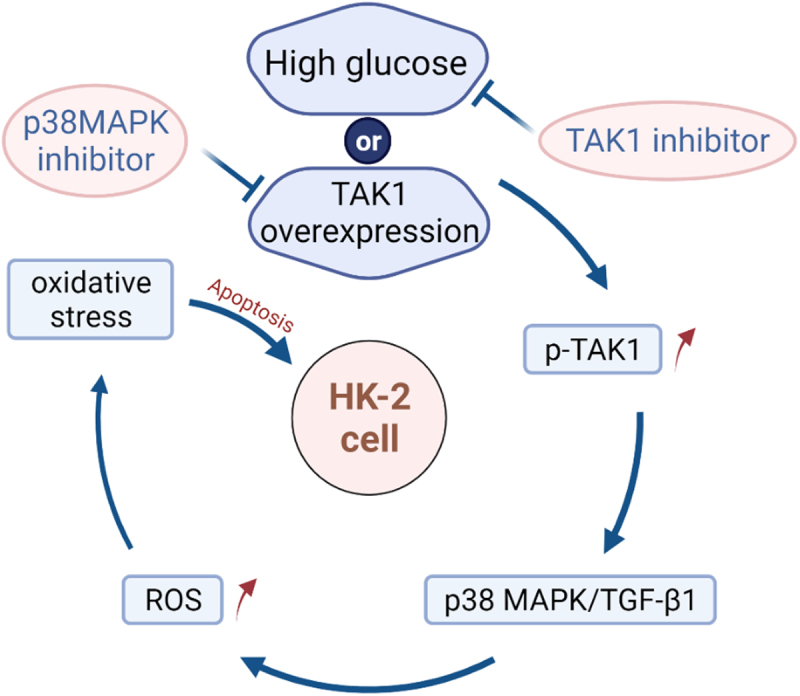

## Introduction

Diabetic nephropathy (DN), as a common diabetic microvascular complication, has gradually become the main cause of end-stage renal disease (ESRD). In the past two decades, the morbidity and mortality of DN in the global population have increased rapidly [1]. The pathogenesis of DN involves multifactorial interactions of metabolic and hemodynamic factors, such as hyperglycemia and advanced glycation end products and the renin–angiotensin system [2]. Hyperglycemia is considered to be a key initiating factor for patients with diabetes to progress to end-stage renal disease, which can activate protein kinase C and induce the production of reactive oxygen species (ROS) [3]. At present, our understanding of the cellular and molecular mechanisms of DN has been significantly improved. However, promising preclinical compounds have failed to show efficacy in humans. Therefore, we need to find a new molecular target for effective treatment of DN.

Apoptosis which functioned as a special way of cell death for further removing the cells not required by the body and keeping the stability of cell number was beneficial to eliminate the cells that were damaged and nonfunctional in the early stage of DN. But, excessive apoptosis of renal cells would happen with the continuous metabolic abnormalities led by diabetes; consequently, it further accelerated the development of diabetic nephropathy by making cell signal transduction level imbalanced and disordering the cell cycle [4]. In terms of the pathogenesis of DN, oxidative stress was also one part in the process except for apoptosis. The damage caused by oxidative stress was also the crucial mechanism causing apoptosis accordingly. In the early stage, the transformation of DN was based on renal tubular lesions; moreover, the damage of renal tubular epithelial cells was the significant factor for DN [5]. So, it was of great significance for the prevention and treatment of DN to carry out research on the oxidative stress and apoptosis in renal tubular cells under the environment with high glucose.

TGF-β-activated kinase 1 (TAK1), which was also called MAP3K7, was actually the member of MAPKKK family engaged in Transforming growth factor-β1 (TGF-β1) signal transduction after being rapidly activated by TGF-β1 and participate in pro-inflammatory signaling pathways [6]. It indicated that TAK1 might be the crucial upstream linkage in the TGF-β1/p38 MAPK (mitogen-activated protein kinase) signal transduction pathway. A large amount of study support the role of TAK1 in inducing cell damage in various stimulant [7]. Another study found that TAK1 may promote the occurrence of diabetic nephropathy by reducing the stability of SnoN protein [8].

TGF-β was categorized as the multiple-functional cell factor that was widely expressed to regulate cell growth, differentiation, apoptosis, repairing of damage in various human tissues. TGF-β1, which functioned as the crucial member in TGF-β super family, was always regarded as one of the strongest fibrogenic factor. Meanwhile, it was also considered as the key cell factor mediating relevant apoptosis accordingly [9,10]. Except for the well-known TGF-β1/Smad signal transduction pathway, MAPK cascade with the mediation of TGF-β1 was also one of the important signal transduction systems. MAPK was divided into three types which were, respectively, ERK, JNK and p38 MAPK. It was verified by various researches that apoptosis of renal tubular epithelial cells and podocyte apoptosis in glomeruli mediated by TGF-β1 were all realized by p38 MAPK pathway. The high expression of TGF-β1 and p38MAPK induced by ROS, which was generated by oxidative stress in diabetic nephropathy would cause apoptosis [11]. Even though a number of researches confirmed that TGF-β1/p38 MAPK had been engaged in renal cell apoptosis and oxidative stress reaction, leading to the relevant renal injury. However, there are no more reports about whether TAK1 affects renal tubular cell apoptosis via TGF-β1/p38 MAPK pathway, which in turn affects the occurrence of DN. 5Z-7-oxozeaenol, a powerful and irreversible inhibitor of TAK1, effectively inhibits TAK1-driven phosphorylation of P38 [12]. SB203580 is a selective inhibitor of p38MAPK, but it does not inhibit the phosphorylation of p38 MAPK by upstream kinase, by inhibiting the catalytic activity of p38MAPK. Instead, p38MAPK plays a role in the activation of MAPKAPK-2 and subsequent phosphorylation of HSP272 [13]. In this study, the above-mentioned two drugs were applied, and the *in vitro* high-glucose model was established to evaluate the effect of TAK1 on renal tubular cell apoptosis in a high-glucose environment, to provide an effective target for the treatment of DN.

## Material and methods

### Apparatus and reagents

CO_2_ incubator (SANYO, Osaka, Japanese), transfer electrophoresis instrument (Liuyi Instrument Factory, Beijing, China); Odyssey FC imager (Li-COR Biosciences, Lincoln City, USA), cyclic variable temperature and heating PCR instrument (Bio-Rad company, Hercules, USA); confocal microscope (LEICA company, Wetzlar, Germany). HK-2 cells (American Type Culture Collection, Rockefeller, USA), 5Z-7-oxozeaenol (Millipore, Massachusetts, USA), SB203580 (APExBIO corporation, Houston, USA), FuGENE6 transfection reagent and TUNEL kit (Promega, Madison, USA), Annexin v-PE/7-AAD Apoptosis kit (BD corporation, New York, USA), ROS kit (Biyuntian Biotechnology, Shanghai, China), Rabbit anti p-TAK1 and t-TAK1 (Abcam, Cambridge, UK), Rabbit anti p38 MAPK and p-p38 MAPK (Cell Signaling, Boston, USA), Rabbit anti Bax, Bcl2 and TGF-β1 (proteintech, Chicago, USA), Horseradish Enzyme Labeled Goat Anti-Rabbit IgG and FITC-Goat Anti-Rabbit IgG (Zhongshan Jinqiao Company, Beijing, China), Trizol (Invitrogen, Carlsbad, USA), Reverse Transcription SysteM and deoxyribonucleoside triphosphate (dNTP) (Promega, Madison, USA), MitoSOX reagent (Sigma, Silicon valley, USA).

### Cell culture and drug stimulation

HK-2 cells were digested and cultured in the DMEM-F12 medium supplemented with 10% fetal bovine serum (FBS) and 1% Penicillin/Streptomycin, and then they were transferred to plates with six holes in accordance with 1 × 10^5^/mL as the concentration. Finally, they were put into incubator with 5% CO_2_ and 37°C for conventional cell cultivation. Synchronization for 24 h was carried out in serum-free medium after HK-2 cells realizes 70% fusion. The cells were divided into four groups respectively: normal glucose group (NG; 5.5 mmol/L glucose), normal glucose + mannitol group (M; 5.5 mmol/L glucose + 24.5 mmol/L mannitol), high glucose group (HG; 30 mmol/L glucose) and high glucose + 5Z-7-oxozeaenol group (HG+5Z; 30 mmol/L glucose+600 nmol/L 5Z-7- oxozeaenol). The cells were collected after being stimulated for 48 h in groups.

### Cell transfection

The cells were divided into five groups, respectively: normal glucose group (NG; 5.5 mmol/l glucose), normal glucose+ mock transfection group (NG+C; 5.5 mmol/L glucose+ 0.5 mg/L pEX-1 empty plasmid), normal glucose+TAK1 overexpression plasmid transfection group (NG+ pEX-3-TAK1; 5.5 mmol/L glucose+0.5 mg/L pEX-3-TAK1), normal glucose + TAK1 overexpression + SB203580 group (NG+ pEX-3-TAK1+ SB203580; 5.5 mmol/L glucose+0.5 mg/L pEX-3-TAK1 + 15 μmol/L SB203580), normal glucose + SB203580 group (NG + SB203580; 5.5 mmol/L glucose+15 μmol/L SB203580). FuGENE6 transfection reagent method was adopted for TAK1 over-expression plasmid (in accordance with the steps in FuGENE6 transfection reagent instructions). For the normal HK-2 cells, they were cultivated in 6-well plate in accordance with the concentration of 1 × 10^5^/mL. Transfection was carried out for cells after undergoing 24 h synchronization in serum-free medium when the cells were fused to 70%. First, 3 μL FuGENE6 transfection reagent was incubated for 5 min in room temperature after being diluted by 97 μL triple-free medium. Then, 1 μg plasmid was added into the mixed liquid of the above-mentioned FuGENE6 and triple-free medium for 15 min of incubation. Afterward, the mixed liquid of the above-mentioned FuGENE6 and plasmid was added into the 6-well plate and cultivated for 6–8 h in 5% CO_2_ incubator with 37°C after being fully mixed. The medium was changed in accordance with different groups and then, the cells were collected after continuous cultivation for 48 h. The following test was carried out.

### Cell apoptosis

The attached cells were digested into cell suspension and then added into EP tube. After it was washed by PBS centrifugation twice, the supernatant fluid was taken out, then each tube was added with 200 μL of 1× binding buffer which was further blown into cell suspension. The mixture of 5 μL Annexin V-PE and 5 μL 7-AAD was added in each tube, then it was incubated at room temperature and far away from sunlight for 15 min after being mixed evenly. Added 300 μL of 1 × binding buffer into each tube, blew and mixed well, then 300 μL of 1× binding buffer was added in each tube after being blown and mixed evenly. Finally, they were further added into flow tube and flow cytometry for observation. The excitation wavelength was 488 mm and the emission wavelength was 530 mm.

### dUTP notch labeling method mediated by terminal deoxynucleotidyl transferase (TUNEL)

Digest HK-2 cells into cell suspension, then inoculate on 8-hole Lab-Tek chamber slide (Nalge Nunc USA), stimulate in groups for 48 h. DeadEndTM fluorescent TUNEL system was used to detect apoptosis according to TUNEL kit instructions. The number of positive stained cells and the total number of cells in each high magnification field were counted under fluorescence microscope.

### Western blotting

Total proteins were extracted from cells. Protein concentration of cells was determined by BCA method. Then, 30 μg proteins were separated by 10% SDS polyacrylamide gel. By means of constant voltage, the voltage in the concentrated glue reached 90 V, after 1 h, the relevant protein sample entered the separation gel with the voltage raised to 120 V. The electric transfer box, which was inside the ice trough, was used to transfer the gel protein and marker to PVDF membrane by electric transferring means. After blocked with 5% skim milk at 37°C for 2 h, the membranes were incubated with antibodies overnight at 4°C. The HRP-IgG II labeled by horseradish peroxidase was added and incubated for 2 h at 37°C in the next day after membrane was washed by the prepared TBST (it was diluted in TBST buffer at the ratio of 1:5000). ECL reagent was added inside after washing membrane and placed in Odyssey FC imaging system for development in this regard. Semi-quantitative analysis was carried out for western bands by adopting ImageJ1.48 software, and the relevant statistical analysis was carried out for average optical density ratio between the target band and β-actin band.

### Immunofluorescence

HK-2 cells were digested by 6-well plates and stimulated for 48 h in groups. Cells were fixed in 4% paraformaldehyde fixed for 30–40 min after washing with precooled PBS for 3 times. Cells were incubated with 0.3% Triton 100 for 10 min at room temperature. Shake PBS 5 min for 3 times, 0.3% Triton 100 incubation for 10 min at room temperature. The cells were then blocked with goat serum at 37°C for 30–40 min and incubated with primary antibody at 4°C overnight. The next day, the cells were rewarmed for 15 min, washed 3 times with PBS and incubated with Fluorescent II antibody without light 37°C for 2 h. The slides were then sealed with DAPI anti-quenching sealant. Photographs were taken with the inverted fluorescence microscope. Quantitative analysis using ImageJ1.48 image processing software, statistical result expressed as average optical density (AOD).

### qRT-PCR

Trizol method was adopted to abstract the total RNA in HK-2 cells, and reverse transcriptase was used to transcribe RNA into cDNA. All the relevant primers were synthesized by Shanghai Shenggong Biotechnology Company (China). The amplification conditions were: pre-denaturation was carried out at 95°C for 10 min and 40 cycles were enlarged within 30s. 18s was regarded as the internal reference correction and method 2^−ΔΔCt^ was adopted to analyze the relative expression of genes. Table 1 presents the primer sequences.

**Table 1.** Primer sequences

RNA	Sequences (5 ‘to 3’)
18S	F: 5’- ACACGGAC- AGGATTACAGA −3’
	R: 5’- GGACATCTAAGGGCATCA CAG −3’
TAK1	F: 5’- ACCGACTCTGCTGTAGCCTCAC-3’
	R: 5’- TCTGTCCTGTCTG- CTGGTCTGTG-3’
TGF-β1	F: 5’-TTGTATGCAGGCCCAGAGGT-3’
	R: 5’- TGGGATCCACCTGCAGCATA −3’
NOX1	F: 5’-ATAGCAGAAGCCGA- CAGG −3’
	R: 5’- CCAGTGAGACCAGCAATG −3’
NOX4	F: 5’- CTCAGCGGAATCAATCAGCTGTG −3’
	R: 5’- AGAGGAACACGACAATCAGCC-TTA −3’
HO-1	F: 5’- CGGGCCAGCAACAAAGTG −3’
	R: 5’- AGTGTAAGGACCCATCGGAGAA −3’

### Detection of intracellular ROS

The fluorescent probe DCHF-DA was used to detect and measure the intracellular ROS. After being digested and subcultured, HK-2 cells were evenly inoculated into 6-well plates, and the medium was discarded after HK-2 cells was stimulated for 48 h in groups. Then, the DCHF-DA staining liquid with final concentration of 10 μmol/L and being diluted with serum-free medium was added. The incubation solution was discarded after it was incubated at 37°C for 30 min in darkness, and then the cells were digested into cell suspension. The cells were centrifuged and washed twice by PBS, and the suspension cells were added into flow tube and detected by flow cytometry. The extent of right deviation of the peak values was assessed by mode value and the latter indicated the fluorescence intensity.

### MitoSOX staining

HK-2 cells were evenly inoculated into 6-well plates after being digested, and the medium was discarded after HK-2 cells were stimulated for 48 h in groups for carrying out MitoSOX staining of living cells. 0.01 mmol/L HBSS was used to wash cells for 3 times, and then the MitoSOX dyestuff with the final concentration of 5 μmol/L was added for further incubation at 37°C for 30 min in darkness. 0.01 mmol/L HBSS was used to wash for 3 times, and the cells were immediately observed and photographed in confocal microscope. Image J software was used to calculate the optical density to quantify staining and to measure the intracellular ROS.

### Statistical analysis

SPSS21.0 statistical software was used for statistics. Experimental data were expressed as mean ± standard deviation (SD). One-way analysis of variance (ANOVA) was applied to comparison among groups. All experiments were repeated three times. *P* < 0.05 indicated that the difference was statistically significant.

## Results

### Inhibition of TAK1 reduces HK-2 cell apoptosis caused by high glucose

**Figure 1.**
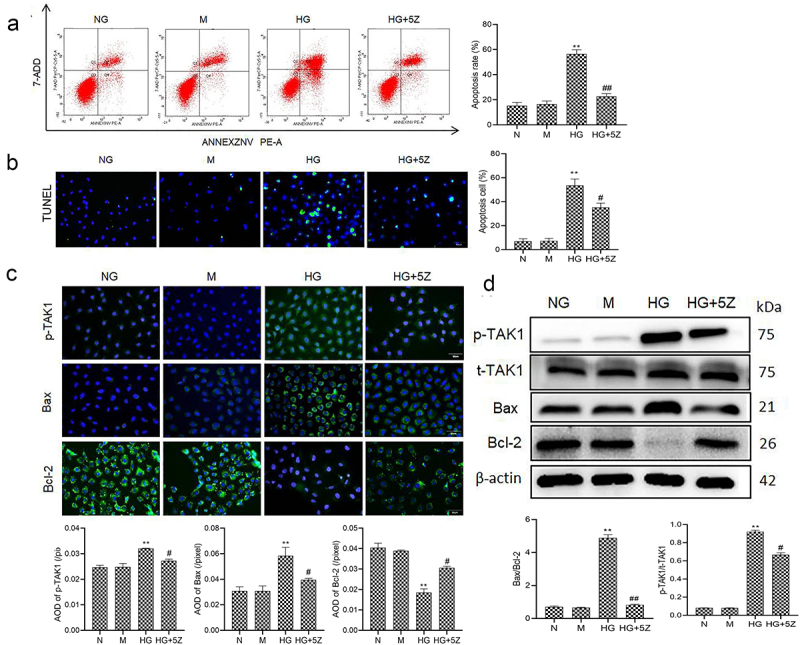
Effect of TAK1 activation on HK-2 cell apoptosis induced by high glucose (a) Apoptosis in different group of HK-2 cells was detected by flow cytometry; (b) Apoptosis in different group of HK-2 cells was detected by TUNEL; (c) The protein expression of p-TAK1, Bax and Bcl-2 was detected by immunofluorescence staining in HK-2 cell; (d) The protein expression of p-TAK1, t-TAK1, Bax, and Bcl-2 was detected by Western blot. All experiments were repeated 3 times. **P < 0.01 *vs*. NG group, ^#^*P* < 0.05 and ^##^*P* < 0.01 *vs*. HG group. NG, 5.5 mmol/l glucose; M, 5.5 mmol/L glucose+24.5 mmol/L mannitol; HG, 30 mmol/L glucose; HG+5Z; 30 mmol/L glucose+600 nmol/L TAK1 inhibitor 5Z-7- oxozeaenol.

First, we studied the effect of TAK1 on the apoptosis of renal tubular cells in DN. The results of flow cytometry and TUNEL showed that high glucose significantly promoted the apoptosis of HK-2 cells compared with NG group (*P* < 0.01). But TAK1 inhibitor 5Z-7-oxozeaenol inhibited high glucose-induced apoptosis of HK-2 cells (*P* < 0.05; Figure 1(a,b)). The results of immunofluorescence showed that compared with NG group, protein expression of p-TAK1 and Bax increased, while the protein expression of Bcl-2 decreased significantly in HG group (*P* < 0.01, Figure 1(c)). The results of Western blot showed that compared with NG group, protein expression ratio of p-TAK1/t-TAK1 and Bax/Bcl-2 increased significantly in HG group (*P* < 0.01, Figure 1(d)). However, 5Z-7-oxozeaenol reversed the expression of the above proteins. Moreover, there was no obvious influence of mannitol on cell apoptosis. It is suggested that TAK1 activation is related to renal tubular cell apoptosis induced by high glucose.

### Inhibition of TAK1 inhibits the activation of TGF-β1/p38MAPK pathway

**Figure 2.**
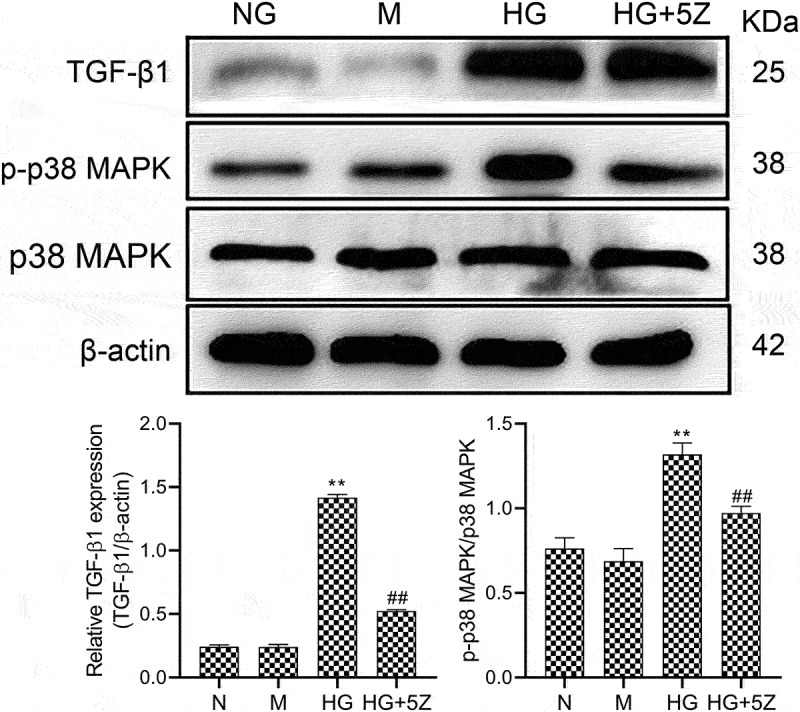
Effects of 5Z-7-oxozeaenol on HG-induced activation of related protein of TGF-β1/p38 MAPK pathway. All experiments were repeated 3 times. **P < 0.01 *vs*. NG group, ^##^*P* < 0.01 *vs*. HG group.

Furthermore, we studied the mechanism of TAK1-induced apoptosis in HK-2 cells. The results showed that compared with NG group, the protein expression of TGF-β1 and ratio of p-p38MAPK/p38MAPK increased significantly in HG group (*P* < 0.01). Compared with HG group, the protein expression of TGF-β1 and ratio of p-p38MAPK/p38MAPK decreased significantly in HG+5Z group (*P* < 0.01, Figure 2). Mannitol had no significant effect on the expression of the above protein. This result indicates that the TGF-β1/p38MAPK signaling pathway is involved in apoptosis mechanism of TAK1 affecting HK-2 cells in high glucose environment.

### Inhibition of TAK1 can inhibit oxidative stress in HK-2 cell

**Figure 3.**
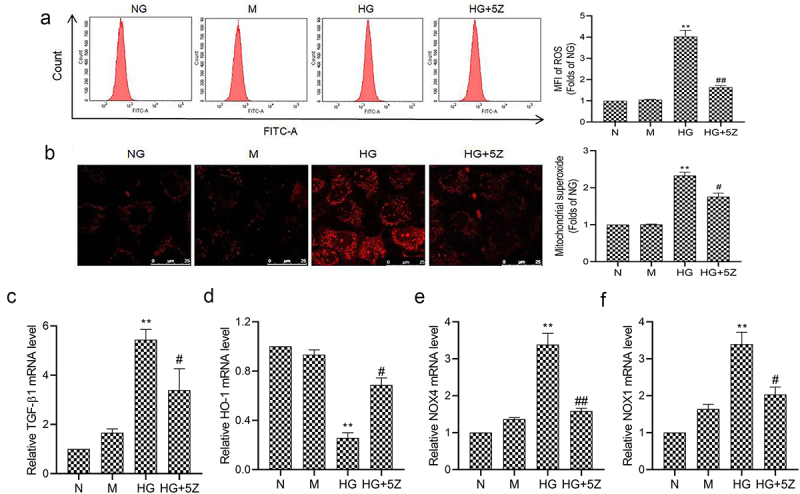
Effects of 5Z-7-oxozeaenol on HG induced oxidative stress in HK-2 cells. (a) Intracellular ROS of HK-2 cells was detected by flow cytometry; (b) Mitochondrial ROS of HK-2 cells was detected by MitoSOX staining; (C, D, E, F) The mRNA expression of TGF-β1, HO-1, NOX4 and NOX1 in HK-2 cells was detected by real-time quantitative PCR. All experiments were repeated three times. **P < 0.01 *vs*. NG group, ^#^*P* < 0.05 and ^##^*P* < 0.01 *vs*. HG group.

The results of flow cytometry showed that compared with NG group, ROS generation of HK-2 cells increased significantly in HG group (*P* < 0.01). And compared with HG group, ROS generation of HK-2 cells decreased significantly in HG+5Z group (*P* < 0.05, Figure 3(a)). And, the results of MitoSOX are consistent with the result of flow cytometry showed that compared with HG group, mitochondrion ROS of HK-2 cells decreased significantly in HG+5Z group (*P* < 0.05, Figure 3(b)). Furthermore, we tested the expression of gene related to oxidative stress, the results showed that compared with NG group, mRNA expression of TGF-β1, NOX1 and NOX4 increased, while HO-1 mRNA expression decreased significantly in HG group (*P* < 0.01; Figure 3(c-f)). However, the expression of the above proteins was reversed in the HG+5Z group. The above results indicated TAK1 is involved in oxidative stress induced by high glucose in HK-2 cells.

### p38MAPK inhibitor SB203580 inhibits the apoptosis induced by TAK1 overexpression in HK-2 cells

**Figure 4.**
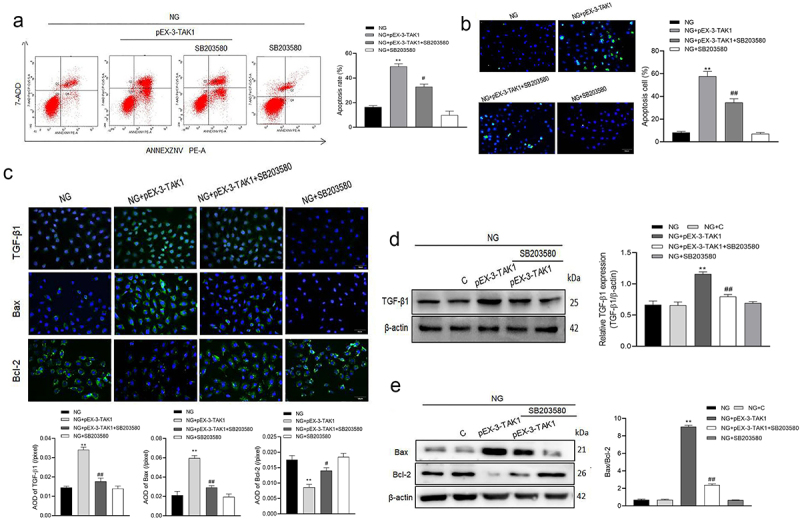
Effects of SB203580 on TAK1 overexpression induced apoptosis in HK-2 cells. (a) Apoptosis was detected by flow cytometry; (b) Apoptosis was detected by TUNEL; (c) The protein expression of TGF-β1, Bax and Bcl-2 in HK-2 cells was detected by immunofluorescence staining. (d) The protein expression of TGF-β1 was detected by Western blot; (e) The protein expression of Bax and Bcl-2 was detected by Western blot; All experiments were repeated 3 times. *P < 0.05 *vs*. NG group, ^#^*P* < 0.05 and ^##^*P* < 0.01 *vs*. NG+ pEX-3-TAK1 group. NG, 5.5 mmol/l glucose; NG+ pEX-3-TAK1, 5.5 mmol/L glucose+0.5 mg/L pEX-3-TAK1; NG+ pEX-3-TAK1+ SB203580; 5.5 mmol/L glucose+0.5 mg/L pEX-3-TAK1 + 15 μmol/L p38MAPK inhibitor SB203580.

Furthermore, we tested the effect of p38MAPK inhibitor SB203580 on apoptosis induced by TAK1 overexpression. The results of flow cytometry and TUNEL showed that after overexpression of TAK1, cell apoptosis increased. However, inhibiting the activation of the p38MAPK pathway on this basis will reverse the effect of TAK1 overexpression (*P* < 0.05, Figure 4(a,b)). The results of immunofluorescence showed that TGF-β1, Bax and Bcl-2 were mainly expressed in cytoplasm of HK-2 cells. Compared with NG+pEX-3-TAK1 group, the protein expression of TGF-β1 and Bax decreased significantly, while the protein expression of Bcl-2 increased significantly in NG+pEX-3-TAK1+ SB203580 group (*P* < 0.05, Figure 4(c)). Western blot showed that the protein expression of TGF-β1 and Bax/Bcl-2 ratio in NG+pEX-3-TAK1+ SB203580 group decreased significantly compared with NG+pEX-3-TAK1 group (*P* < 0.05, Figure 4(d,e)). Furthermore, the results explain the role of p38MAPK pathway in the influence of TAK1 on apoptosis in high glucose environment.

### p38MAPK inhibitor SB203580 inhibits the ROS production induced by TAK1 overexpression in HK-2 cells

**Figure 5.**
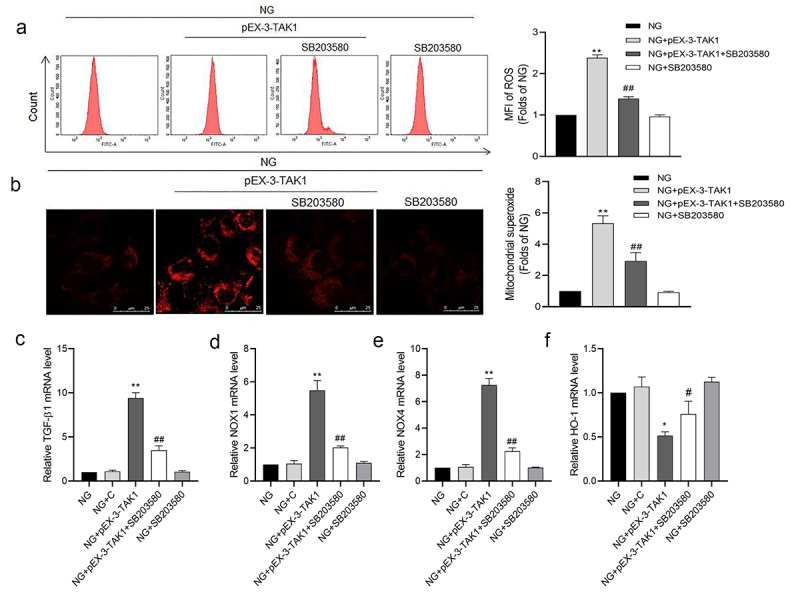
Effects of p38 MAPK inhibitor SB203580 on TAK1 overexpression induced oxidative stress in HK-2 cells. (a) Intracellular ROS was detected by flow cytometry; (b) Mitochondrial ROS was detected by MitoSOX staining. (C, D, E, F) The mRNA expression of TGF-β1, NOX1, NOX4 and HO-1 in HK-2 cells was detected by real time quantitative PCR. All experiments were repeated three times. *P < 0.05 and ** *P* < 0.01 *vs*. NG group, ^#^*P* < 0.05 and ^##^*P* < 0.01 *vs*. NG+pEX-3-TAK1 group. NG+C, 5.5 mmol/L glucose+ 0.5 mg/L pEX-1 empty plasmid.

The results of flow cytometry showed that compared with NG group, ROS generation of HK-2 cells increased significantly in NG+pEX-3-TAK1 group (*P* < 0.05). But compared with NG+pEX-3-TAK1 group, ROS generation of HK-2 cells decreased significantly in NG+pEX-3-TAK1 + SB203580 group (*P* < 0.05, Figure 5(a)). The results of MitoSOX staining are consistent with the result of flow cytometry (*P* < 0.05, Figure 5(b)). The results of qRT-PCR showed that compared with NG group, mRNA expression of TGF-β1, NOX1 and NOX4 increased, while HO-1 mRNA expression decreased significantly in NG+pEX-3-TAK1 group (*P* < 0.05). However, SB203580 reverses the expression of genes above the NG+pEX-3-TAK1 group (*P* < 0.05, Figure 5(c-f)).

## Discussion

It is verified by various studies that the renal tubular atrophy led by apoptosis and shedding of renal tubular cells will directly cause the declining of renal functions, which can lead to DN. If the disease cannot be controlled in time, the latter will eventually turn deterioration into ESRD. Moreover, the deposition of extracellular matrix and renal fibrosis will also be promoted accordingly, which further intensifies the damage of renal structure and functions. Under the condition of high glucose *in vitro*, the apoptosis of renal tubular epithelial cells in DN patients increased [14]. In this regard, the extent of apoptosis in renal tubular epithelial cells is closely relevant to DN development and prognosis [15]. Oxidative stress refers to the imbalance between the generation of ROS and antioxidant substance, which will lead to tissue damage. The excessive ROS may be generated in the high glucose state. Then, apoptosis will be triggered by a series of stress sensitive signaling pathways, which leads to cell damage, abnormality of renal function and histological changes [16,17]. So, when it comes to the excessive generation of ROS, it is considered as one of the significant factors for cell damage and the starting of diabetic nephropathy. In this study, we found the high glucose significantly induced oxidative stress in HK-2 cells and promoted apoptosis.

In addition, the results of this study confirmed that high glucose is able to raise the protein expression of p-p38MAPK and TGF-β1 in HK-2 cells. It has already been proved by the previous researches that TGF-β1 plays a crucial part in the apoptosis process of renal tubular epithelial cells, and there are two ways for downstream signal transduction of TGF-β1: Smads signal transduction pathway and MAPK signal transduction pathway [10]. Dai et al. [10] believed that TGF-β1 leads to the apoptosis of renal tubular epithelial cells by MAPK pathway while not by Smads pathway. And Wu et al. [18] revealed that the apoptosis of glomerular podocytes in mouse mediated by TGF-β1 is realized by p38MAPK pathway. p38MAPK signaling pathway is considered as a crucial branch for MAPK signaling pathway, as well as the intersection point for cell signal transduction. In one aspect, TGF-β1 is able to play its role by activating p38 MAPK in the upstream of the signaling pathway. In another aspect, the expression of TGF-β1 can be decreased by inhibiting p38 in multiple nephrotic models [19,20]. On such basis, it is proved that TGF-β1 can be the downstream product of p38MAPK signaling pathway, and there is obvious mutual interaction between TGF-β1 and p38MAPK. Jung et al. [21] proved that inhibiting p38MAPK pathway by FR167653 can improve apoptosis of mesangial cells induced by high glucose, which indicates that p38MAPK signaling pathway is closely relevant to apoptosis of renal cells.

MAPK cascade is a continuous protein kinase chain, as well as the activation process of MAPKKK-MAPKK-MAPK cascade by cascade, so only after MAPK is activated can it cause various biological effects accordingly. TAK1 is also called MAP3K7, which is regarded as the only member of MAPKKK protein family engaged in TGF-β1 signal transduction. TAK1 is able to phosphorylate MAPK, kinases MKK4 and MKK3/6, then these kinases will activate JNK and p38 MAPK, respectively, so their function as the important regulation factors in upstream MAPK pathway. However, there is still considerable controversy for its effect on apoptosis. TAK1 can only be activated after being bound with the specific TAK1 binding protein (TAB1.2.3). Moreover, TAK1-Table 1 complex is indispensable for normal embryonic development and morphological development. Therefore, the inactivation of Table 1 gene may possibly lead to developmental defects of major organs and embryo death [22]. It is revealed by relevant research that knocking down the expression of TAK1 can promote apoptosis in various cell types in vivo or in vitro [23,24]. The absence of TAK1 gene also leads to apoptosis twice than that of obstructive kidney model of unilateral ureteral obstruction [25], which indicates that TAK1 is an essential factor to prevent apoptosis and keep cells survive. But, the pathological state will be caused by the continuous and excessive activation of TAK1. It is of great importance to lower the TAK1 activity for the prevention of excessive TGF-β1 reaction and reduction of cell damage. Wu et al. [26] revealed that the improvement of renal interstitial fibrosis in mice of ischemia-reperfusion with propofol is actually realized by lowering the expression of TAK1 and inhibiting apoptosis in the early stage. This has similarity with the results of the present study. We then found that high glucose induced the expression of TAK1 in HK-2 cells. And, TAK1 Inhibitor 5Z-7-oxozeaenol is able to obviously decrease the oxidative stress and apoptosis of HK-2 cells induced by high glucose. At the same time, it can also inhibit activation of p38MAPK and the expression of TGF-β1 induced by high glucose. Furthermore, p38MAPK inhibitor SB203580 could decrease the expression of TGF-β1, block oxidative stress and apoptosis induced by TAK1 overexpression in HK-2 cells. As hinted, the protection of 5Z-7-oxozeaenol for HK-2 cells with the stimulation of high glucose may partly inhibit TAK1/ p38MAPK/ TGF-β1 pathway and reduce oxidative stress and apoptosis of cells.

It is showed by the above-mentioned different results that TAK1 possibly plays dual roles in promoting apoptosis and anti-apoptosis in accordance with different environmental backgrounds, tissue and cell types.

## Conclusion

Our research suggests that TAK1 can maintain low phosphorylation level in normal renal tubular epithelial cells. High glucose will promote the phosphorylation of TAK1, which thereby activate p38MAPK/TGF-β1 pathway, induce oxidative stress, generate excessive ROS and induce apoptosis. In this regard, TAK1 will help to reduce the apoptosis of renal tubular epithelial cells induced by high glucose.

## Highlights

- High glucose up-regulates the p-TAK1 expressions.
- TAK1 inhibitor 5Z-7-oxozeaenol 5Z-7-oxozeaenol inhibits high glucose-induced oxidative stress in HK2 cells.
- 5Z-7-oxozeaenol inhibits high glucose-induced apoptosis in HK2 cells.
- Inhibition of TAK1 expression blocks the p38 MAPK/TGF-β1 signaling pathway.

## References

[1] Heerspink HJL, Parving HH, Andress DL, et al. Atrasentan and renal events in patients with type 2 diabetes and chronic kidney disease (SONAR): a double-blind, randomised, placebo-controlled trial. Lancet. 2019;393(10184):1937–1947. 30995972 10.1016/S0140-6736(19)30772-X

[2] Kumar Pasupulati A, Chitra PS, Reddy GB. Advanced glycation end products mediated cellular and molecular events in the pathology of diabetic nephropathy. Biomol Concepts. 2016;7(5–6):293–309. 27816946 10.1515/bmc-2016-0021

[3] Volpe CMO, Villar-Delfino PH, Dos Anjos PMF, et al. Cellular death, reactive oxygen species (ROS) and diabetic complications. Cell Death Dis. 2018;9(2):119. 29371661 10.1038/s41419-017-0135-z PMC5833737

[4] Priante G, Gianesello L, Ceol M, et al. Cell Death in the Kidney. Int J Mol Sci. 2019;20(14):3598. 10.3390/ijms20143598 PMC6679187 31340541

[5] Lee HJ, Lee DY, Mariappan MM, et al. Hydrogen sulfide inhibits high glucose-induced NADPH oxidase 4 expression and matrix increase by recruiting inducible nitric oxide synthase in kidney proximal tubular epithelial cells. J Biol Chem. 2017;292(14):5665–5675. 28188286 10.1074/jbc.M116.766758 PMC5392562

[6] Ajibade AA, Wang HY, Wang RF. Cell type-specific function of TAK1 in innate immune signaling. Trends Immunol. 2013;34(7):307–316. 23664135 10.1016/j.it.2013.03.007

[7] Sakurai H. Targeting of TAK1 in inflammatory disorders and cancer. Trends Pharmacol Sci. 2012;33(10):522–530. 22795313 10.1016/j.tips.2012.06.007

[8] Wang Y, Mao Y, Zhang X, et al. TAK1 may promote the development of diabetic nephropathy by reducing the stability of SnoN protein. Life Sci. 2019;228:1–10. 31028803 10.1016/j.lfs.2019.04.058

[9] Miyajima A, Chen J, Lawrence C, et al. Antibody to transforming growth factor-beta ameliorates tubular apoptosis in unilateral ureteral obstruction. Kidney Int. 2000;58(6):2301–2313. 11115064 10.1046/j.1523-1755.2000.00414.x

[10] Dai C, Yang J, Liu Y. Transforming growth factor-beta1 potentiates renal tubular epithelial cell death by a mechanism independent of Smad signaling. J Biol Chem. 2003;278(14):12537–12545. 12560323 10.1074/jbc.M300777200

[11] Jiang M, Zhang H, Zhai L, et al. ALA/LA ameliorates glucose toxicity on HK-2 cells by attenuating oxidative stress and apoptosis through the ROS/p38/TGF-β(1) pathway. Lipids Health Dis. 2017;16(1):216. 29145851 10.1186/s12944-017-0611-6 PMC5691398

[12] Wu J, Powell F, Larsen NA, et al. Mechanism and In Vitro Pharmacology of TAK1 Inhibition by (5Z)-7-Oxozeaenol. ACS Chem Biol. 2013;8(3):643–650. 23272696 10.1021/cb3005897

[13] Xia A, Li Y, Li N, et al. Roles of MAPKAPK-2 and HSP27 in the reduction of renal ischemia-reperfusion injury by ischemic postconditioning in rats. Int Urol Nephrol. 2014;46(7):1455–1464. 24927932 10.1007/s11255-014-0748-4

[14] Yu R, Zhang Y, Lu Z, et al. Long-chain non-coding RNA UCA1 inhibits renal tubular epithelial cell apoptosis by targeting microRNA-206 in diabetic nephropathy. Arch Physiol Biochem. 2019;1–9. 10.1080/13813455.2019.1673431. 31608712

[15] Guo Y, Xie X, Zhao Y, et al. Calcitriol attenuates renal tubular epithelial cells apoptosis via inhibiting p38MAPK signaling in diabetic nephropathy. Acta Diabetol. 2020;57(11):1327–1335. 32556611 10.1007/s00592-020-01554-0

[16] Rochette L, Zeller M, Cottin Y, et al. Diabetes, oxidative stress and therapeutic strategies. Biochim Biophys Acta. 2014;1840(9):2709–2729. 24905298 10.1016/j.bbagen.2014.05.017

[17] Matsuda M, Shimomura I. Increased oxidative stress in obesity: implications for metabolic syndrome, diabetes, hypertension, dyslipidemia, atherosclerosis, and cancer. Obes Res Clin Pract. 2013;7(5):e330–41. 24455761 10.1016/j.orcp.2013.05.004

[18] Wu DT, Bitzer M, Ju W, et al. TGF-beta concentration specifies differential signaling profiles of growth arrest/differentiation and apoptosis in podocytes. J Am Soc Nephrol. 2005;16(11):3211–3221. 16207831 10.1681/ASN.2004121055

[19] Grygielko ET, Martin WM, Tweed C, et al. Inhibition of gene markers of fibrosis with a novel inhibitor of transforming growth factor-beta type I receptor kinase in puromycin-induced nephritis. J Pharmacol Exp Ther. 2005;313(3):943–951. 15769863 10.1124/jpet.104.082099

[20] Nishida M, Okumura Y, Sato H, et al. Delayed inhibition of p38 mitogen-activated protein kinase ameliorates renal fibrosis in obstructive nephropathy. Nephrol Dial Transplant. 2008;23(8):2520–2524. 18515792 10.1093/ndt/gfn309

[21] Jung DS, Li JJ, Kwak SJ, et al. FR167653 inhibits fibronectin expression and apoptosis in diabetic glomeruli and in high-glucose-stimulated mesangial cells. Am J Physiol Renal Physiol. 2008;295(2):F595–604. 18524857 10.1152/ajprenal.00624.2007

[22] Komatsu Y, Shibuya H, Takeda N, et al. Targeted disruption of the Tab1 gene causes embryonic lethality and defects in cardiovascular and lung morphogenesis. Mech Dev. 2002;119(2):239–249. 12464436 10.1016/s0925-4773(02)00391-x

[23] Stambe C, Nikolic-Paterson DJ, Hill PA, et al. Mitogen-activated protein kinase activation and cell localization in human glomerulonephritis: correlation with renal injury. J Am Soc Nephrol. 2004;15(2):326–336. 14747379 10.1097/01.asn.0000108520.63445.e0

[24] Omori E, Matsumoto K, Zhu S, et al. Ablation of TAK1 upregulates reactive oxygen species and selectively kills tumor cells. Cancer Res. 2010;70(21):8417–8425. 20959492 10.1158/0008-5472.CAN-10-1227 PMC2970664

[25] Ma FY, Tesch GH, Ozols E, et al. TGF-β1-activated kinase-1 regulates inflammation and fibrosis in the obstructed kidney. Am J Physiol Renal Physiol. 2011;300(6):F1410–21. 21367917 10.1152/ajprenal.00018.2011

[26] Wu H, Zhou J, Ou W, et al. TAK1 as the mediator in the protective effect of propofol on renal interstitial fibrosis induced by ischemia/reperfusion injury. Eur J Pharmacol. 2017;811:134–140. 28603043 10.1016/j.ejphar.2017.06.009

